# Calciphytoliths (calcium oxalate crystals) analysis for the identification of decayed tea plants (*Camellia sinensis* L.)

**DOI:** 10.1038/srep06703

**Published:** 2014-10-24

**Authors:** Jianping Zhang, Houyuan Lu, Linpei Huang

**Affiliations:** 1 Key Laboratory of Cenozoic Geology and Environment, Institute of Geology and Geophysics, Chinese Academy of Sciences, 100029, Beijing, China; 2 Key Laboratory of Plateau Lake Ecology and Global Change, College of Tourism and Geography, Yunnan Normal University, Kunming, Chenggong 650500, China

## Abstract

The history of tea is poorly known, mainly due to the questionable identification of decayed tea plants in archaeological samples. This paper attempts to test the utility of calciphytoliths (calcium oxalate crystals) for the identification of tea in archaeological samples. It provides the first survey of the macropatterns of calciphytoliths in several species of Theaceae and common non-Theaceae plants. Crystals were extracted from 45 samples of tea, Theaceae and common non-Theaceae plants, and detected microscopically between crossed polarizers. In tea plants, druse and trichome base are the most distinctive crystals. Druses have the smallest diameter (11.65 ± 3.64 μm), and trichome bases have four distinctive straight and regular cracks, similar to a regular extinction cross. The results provide morphological criteria for distinguishing tea from other plants, specifically the presence of identifiable druses together with calcified trichome bases. The implications are significant for understanding the history of tea and plant exploitation, especially for plants for which the preservation of macrofossils is poor.

Calciphytoliths (calcium oxalate crystals) are microscopic mineralized bodies that have been observed in rocks, soil, and among multiple members of all five kingdoms (Monera, Protista, Fungi, Plantae, and Animalia)^1^. They are estimated to occur in at least 75% of angiosperms, being present in most tissues and organs, such as roots, bark, stems, leaves, flowers, fruits, and seeds, and they exhibit various sizes and shapes^2^,^3^. The most common morphologies in plants are druses (a spherical aggregate of individual crystals), styloids (elongated crystals with pointed or ridged ends), raphides (needle-shaped crystals occurring in bundles of many crystals per cell), prisms and crystal sands (a mass of many tiny, individual crystals in a single cell)^4^,^5^,^6^. Druses are clusters of crystals while the others are individual crystals. These crystals are released into sediments when plant tissues are decayed or burned. Released calciphytoliths thus become microfossils of the plants that produce them.

Usually, the morphology of a crystal, as well as its spatial distribution, called the crystal macropattern, is characteristic of specific taxa at any level from family to individual species within a genus^2^,^3^,^7^,^8^,^9^,^10^,^11^,^12^. In addition, the specific features of calciphytoliths permit the identification of different plant parts from both dietary and non-dietary plants^13^, including a great number of Chinese medicinal herbs^14^, and have been successfully used to document important aspects of the prehistoric diet^15^,^16^,^17^,^18^,^19^, especially in the case of plants that have seldom been preserved in the macrobotanical and silica-phytolith record. Calciphytoliths play an important role in plant exploration research, but work to date has only provided limited morphological investigation in dietary plants.

Tea, a product made up from the leaf and bud of the plant *Camellia sinensis* L*.*, has been consumed by humans for thousands of years and it is the second most consumed beverage in the world after water, well ahead of coffee, beer, wine and carbonated soft drinks^20^. The tea plant, *Camellia sinensis* L., belongs to section *Thea*, genus *Camellia*, family Theaceace. Generally, it is considered to have originated in southwestern China, and has been consumed in increasing quantities over the past 2000 years^21^,^22^.

However, our knowledge of the history of tea in China, especially in Neolithic China, is incomplete^21^,^23^,^24^,^25^, mainly due to the limited occurrence of macro-remains. Unlike cereal grains, tea decays rapidly because of the abundant moisture in the leaves^26^,^27^, thus making it difficult to distinguish tea in archaeological samples. This paper reports the first attempt to investigate the occurrence of calcium oxalate crystals of tea and to determine if the crystals of the leaves can be used as an effective tool to distinguish tea from other plant remains. The implications of the findings are significant for understanding the history of tea and plant exploitation, especially where plant macrofossil preservation is poor.

## Results

### General observations

**Figure 1:**
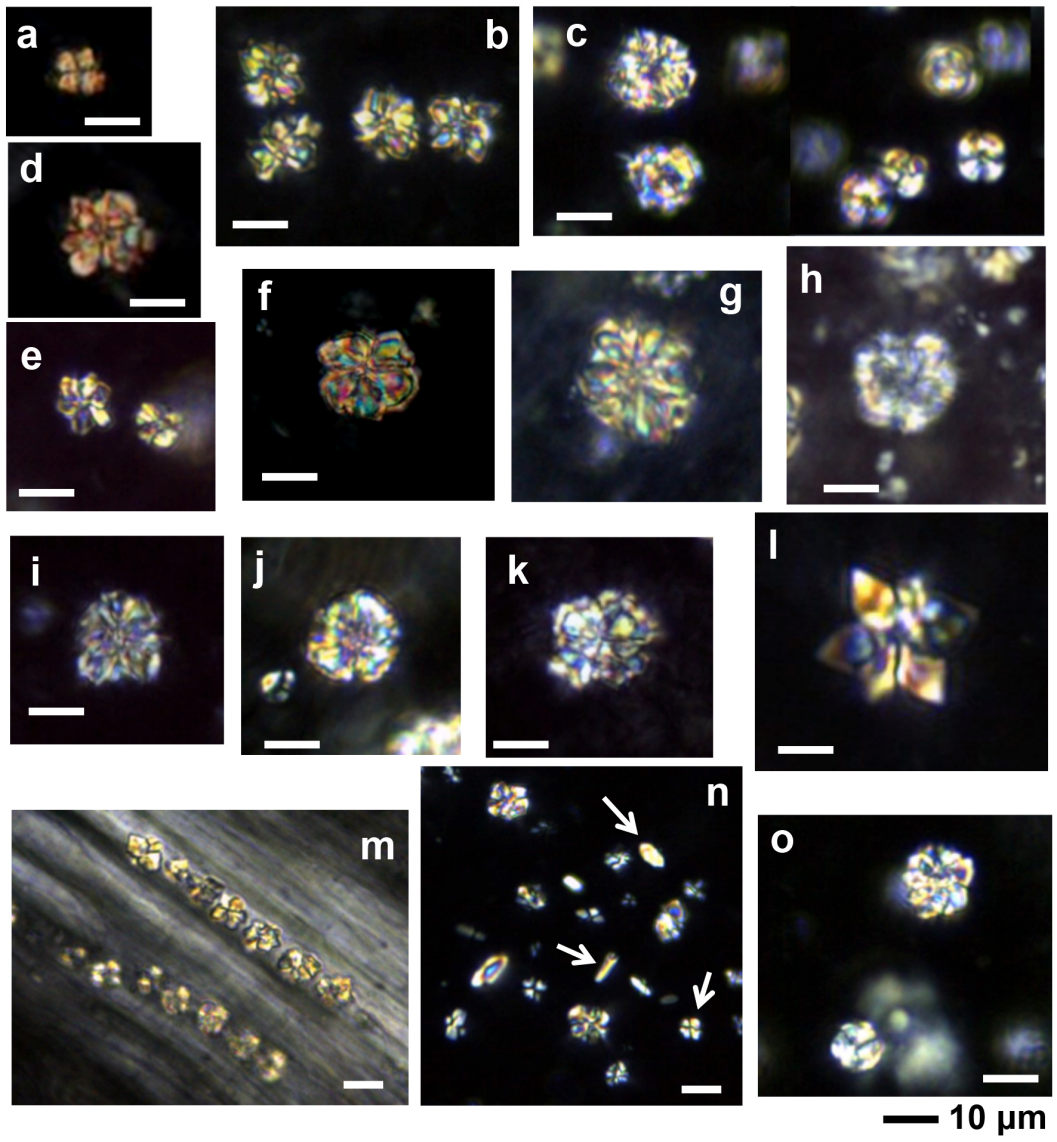
Druses in *Camellia sinensis* L. a) Longjing; b, m) Liu'anguapian; c) Xinyangmaojian; d) Tieguanyin; e) An'huihoukui; f) Yeshengdayecha; g) Mengdingcha; h) Sichuanbaihao; i) Fujianlvcha; j) Sichuanqueshe; k) Baihaoyinzhen; l) Fujianbaicha; n, o) Zhuyeqing. The white arrows indicate secondary crystals.

**Figure 2:**
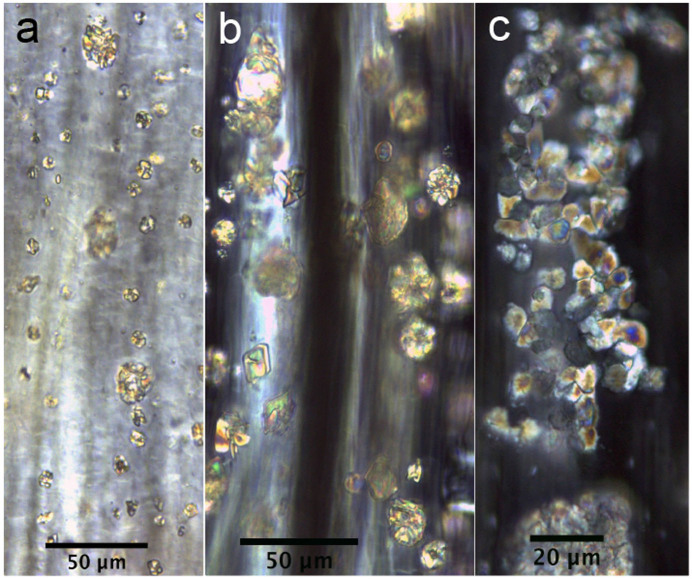
Intermediate crystals in vascular bundles. a) *Camellia sinensis* L. Mengdingcha; b) *Prunus mahaleb* var. *cupaniana*; c) *Morus australis*.

**Figure 3:**
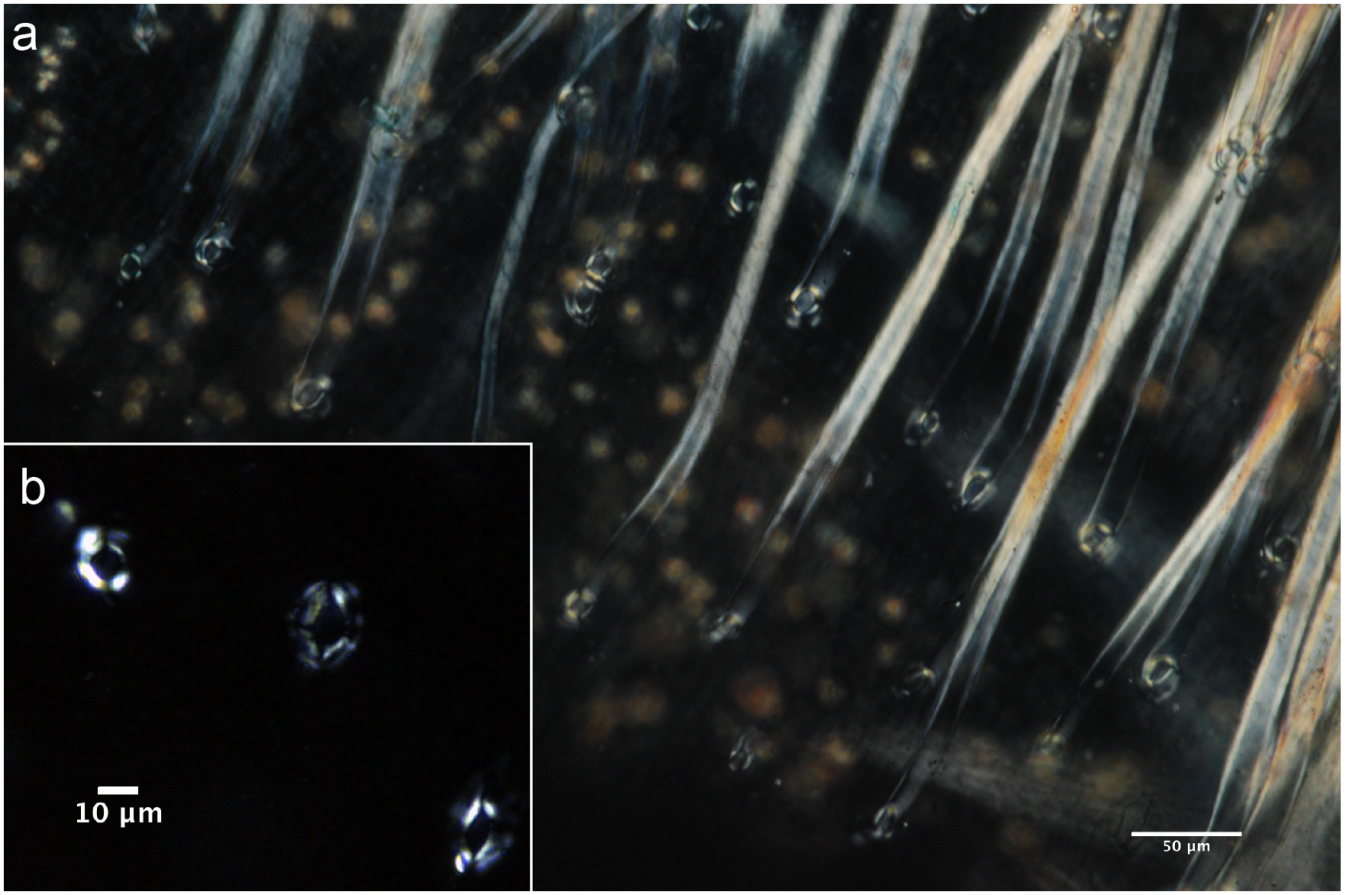
Calcified trichome bases in *Camellia sinensis* L. a) Low magnification overview of calcified trichome and the bases; b) a higher magnification view of a single base.

**Figure 4:**
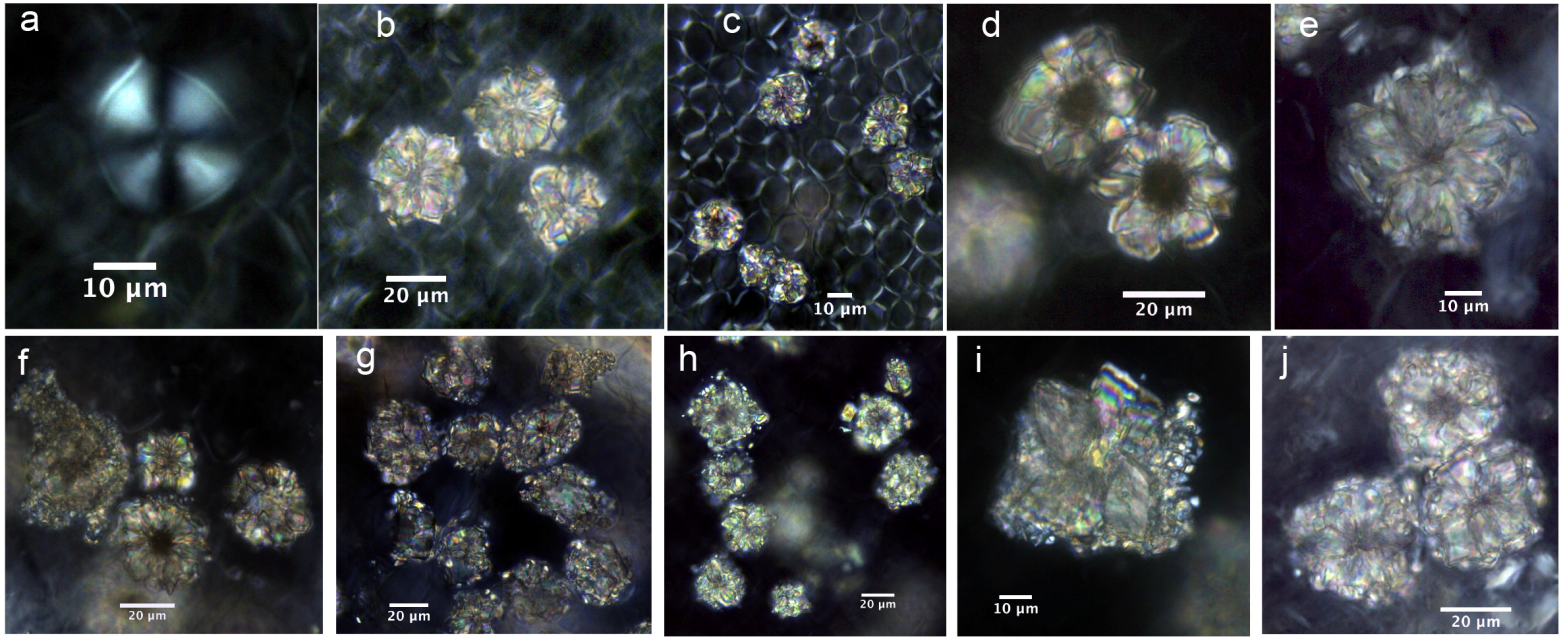
The major types of calcium oxalate crystals in Theaceae plants. a) calcified trichome base in *Camellia synaptica* S.; druses in b) *Camellia synaptica* S.; c) *Camellia impressinervis*; d) *Sladenia celastrifolia* Kurz; e) *Camellia crassipes*; f) *Camellia reticulate* L. ‘Snow Elegans'; g) *Camellia japonica* L. ‘Sun Song'; h) *Camellia sasanqua* T. ‘Read Jade'; i) *Camellia reticulata* L.‘Baozhucha'; j) *Camellia costei* Cevl.

**Figure 5:**
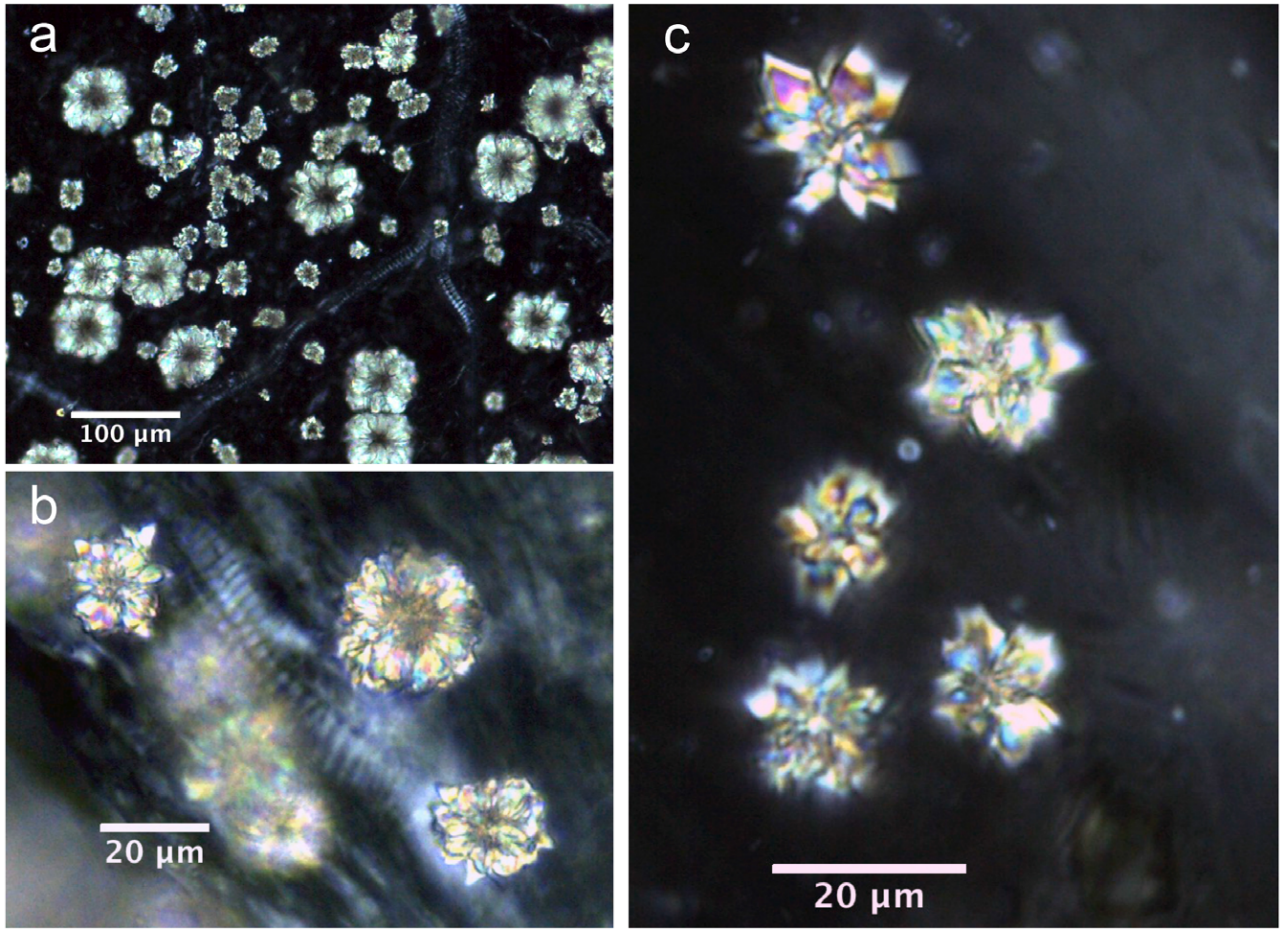
Druses in non-Theaceae plants. a) *Dianthus chinensis* L.; b) *Portulaca oleracea* L.; c) *Malva sinensis*.

**Figure 6:**
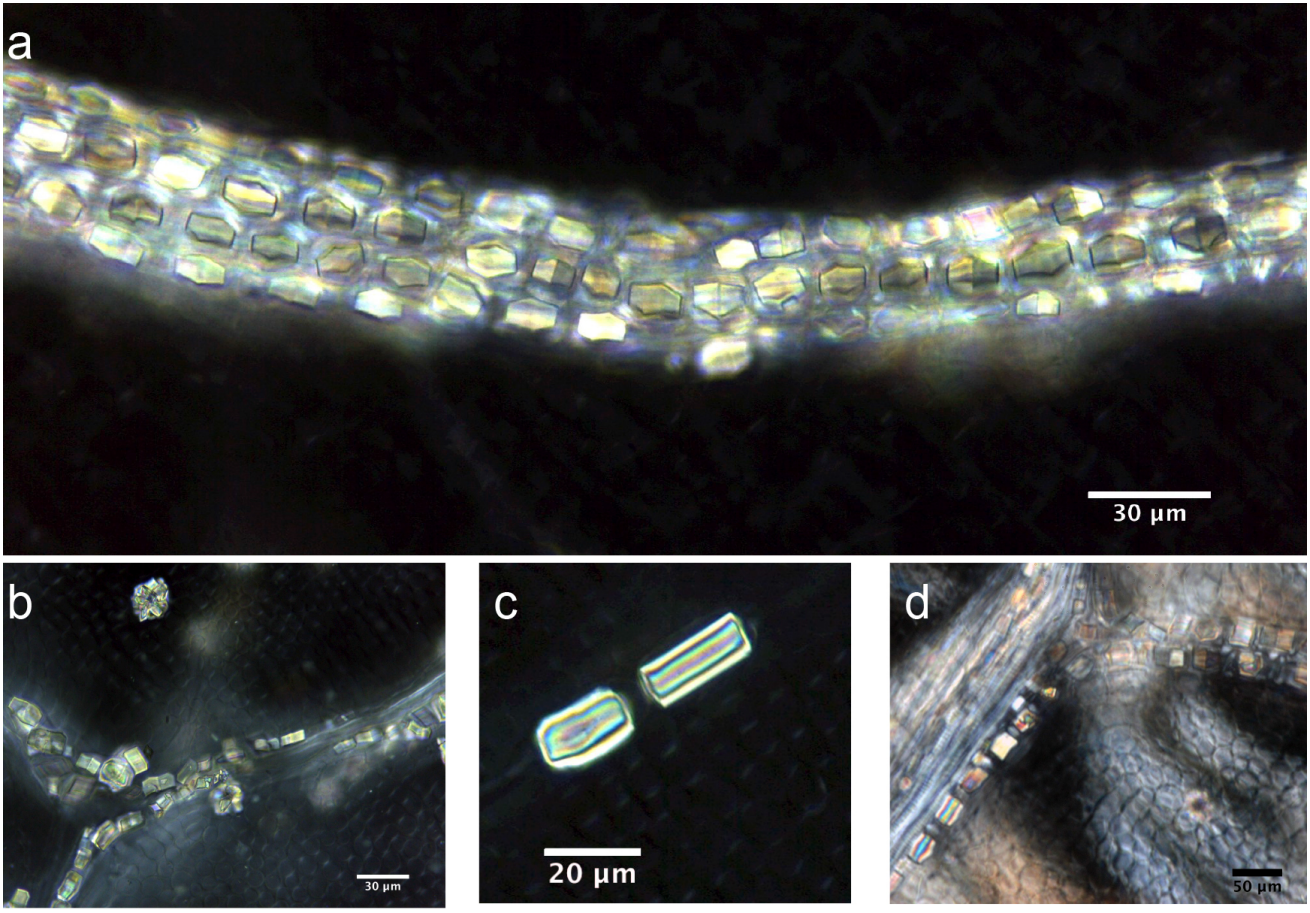
Prisms in non-Theaceae plants. a) *Lespedeza bicolor* Turcz.; b) *Zelkova schneideeeriana* Hand.-Mazz.; c) *Tilia tuan* Szyszyl.; d) *Salix ernesti*.

**Figure 7:**
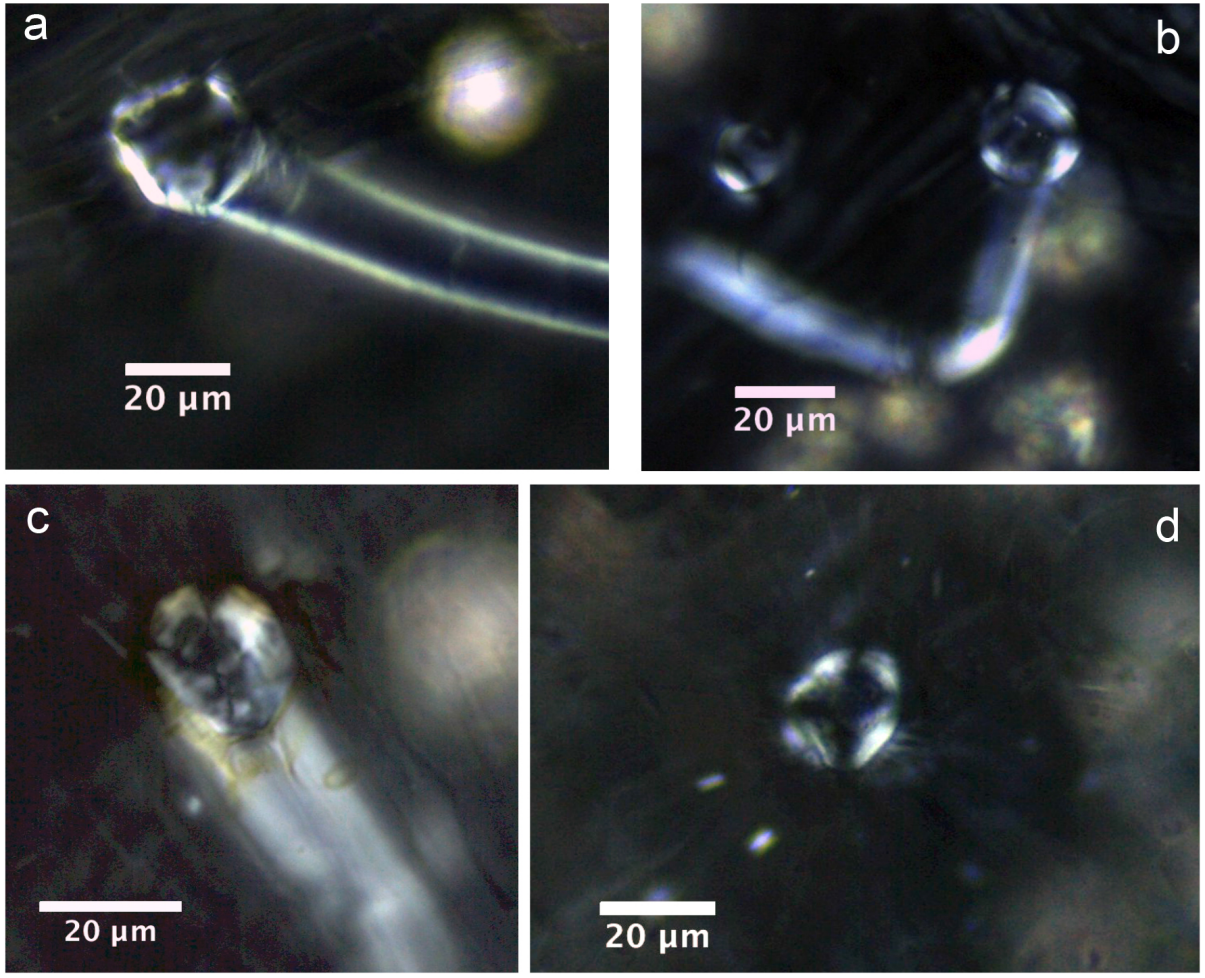
Calcified trichome bases in non-Theaceae plants. a) *Prunus mahaleb* var. *cupaniana*; b) *Corylus heterophylla*; c) *Betula delavayi*; d) *Morus australis*.

**Figure 8:**
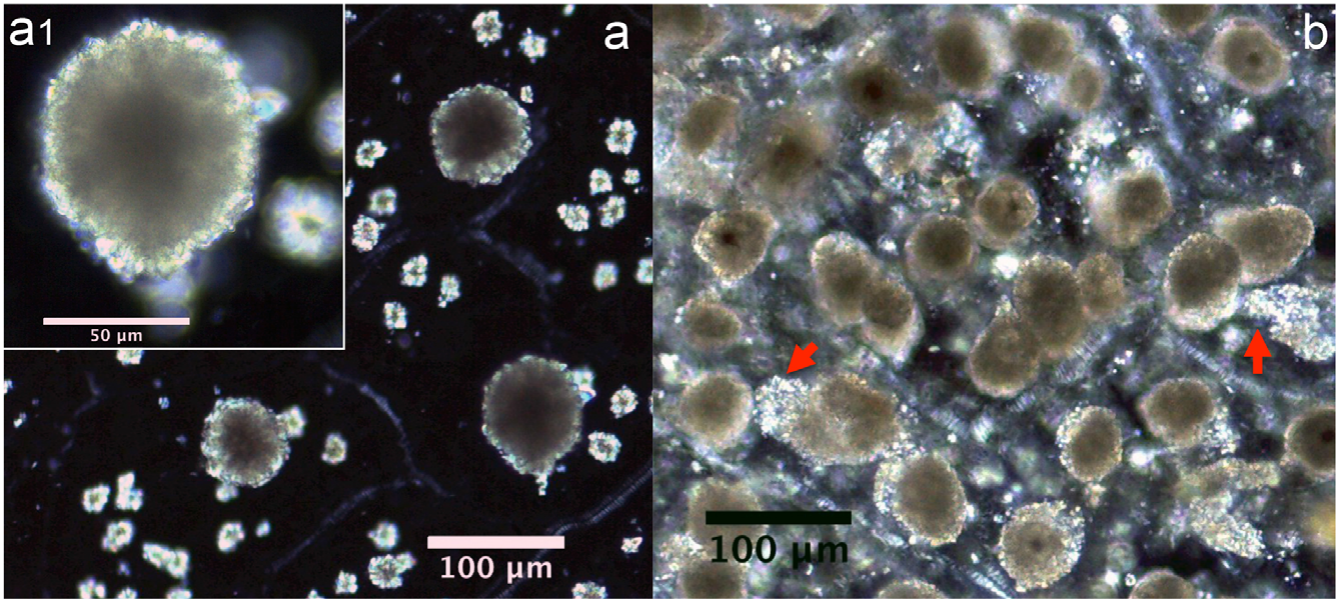
Concretions in non-Theaceae plants. a) *Chenopodium album*; a1) a higher magnification overview of single concretion; b) *Lycium* sp. The red arrows point to crystal sands.

**Figure 9:**
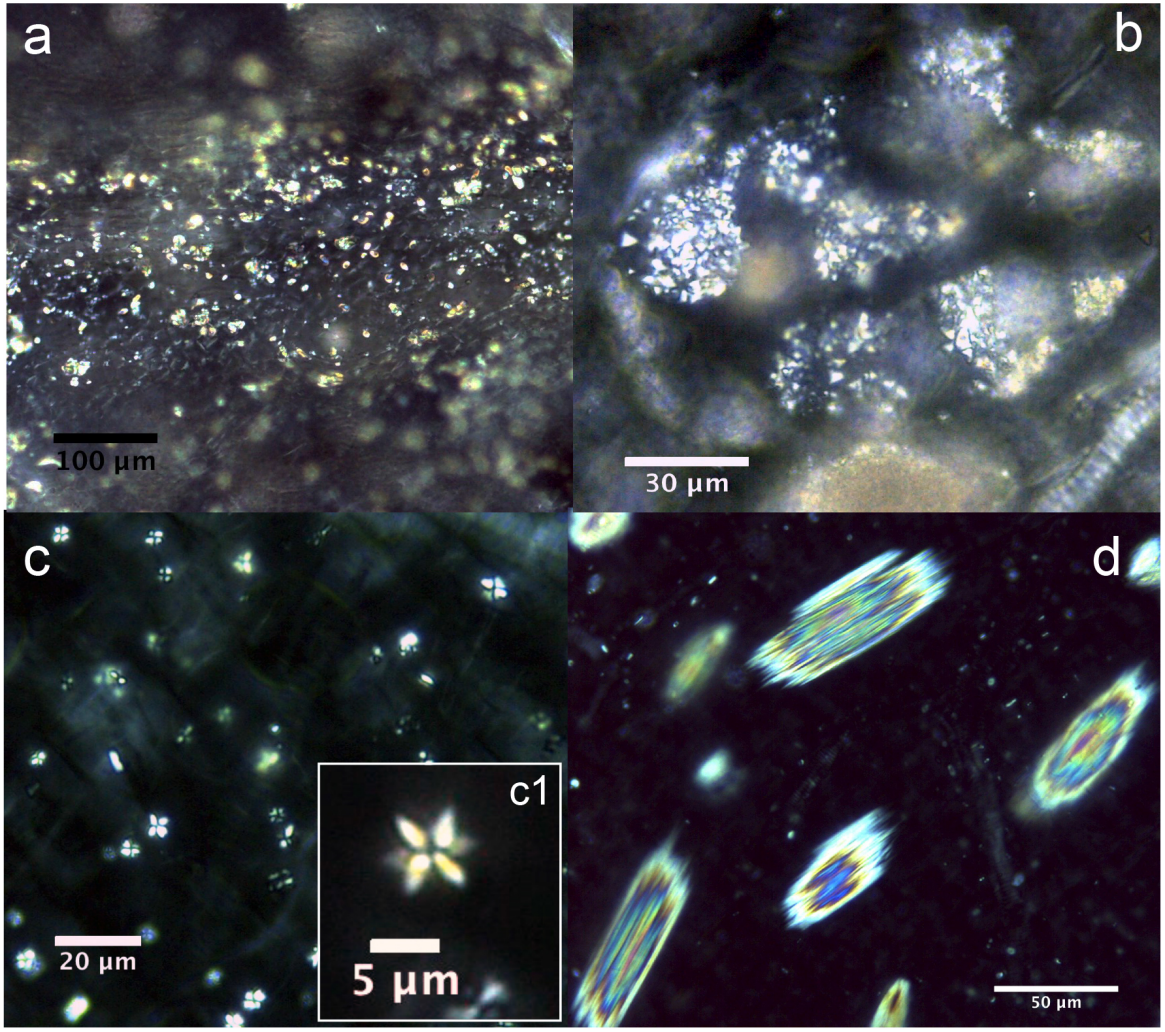
Crystal sands and secondary crystals in non-Theaceae plants. a) *Cynanchum komarovii* Al. Iljinski; b) *Lycium* sp.; c) *Magnolia sieboldii* K. Koch; c1) a higher magnification overview of star-like crystal aggregates; d) *Epilobium tibetanum* Hausskn.

**Figure 10:**
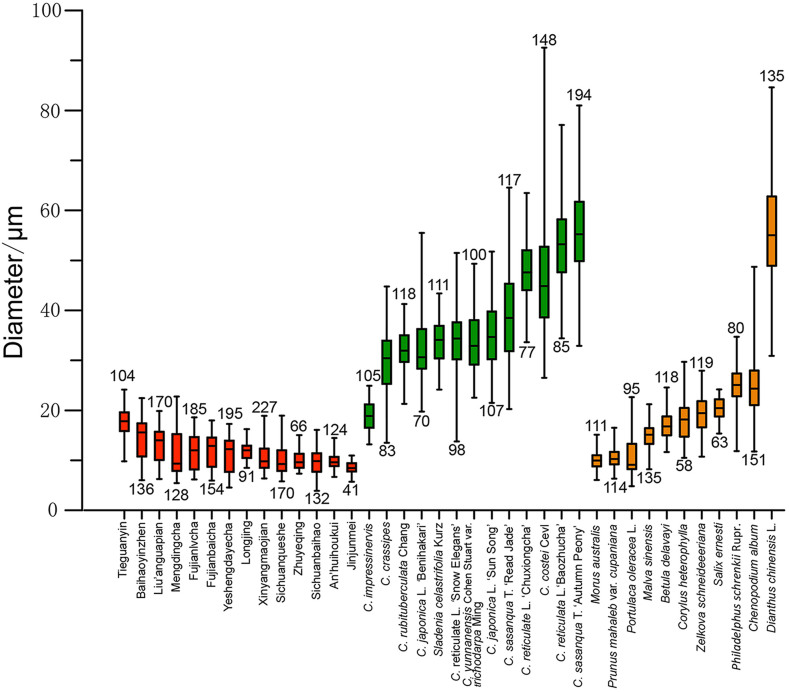
Box plots of druse sizes in the investigated plants. Red bars refer to *Camellia sinensis* L. plants; green bars to non-Theaceae plants; and orange bars to non-Theaceae plants. The number at both ends of each box is the number of observations.

**Figure 11:**
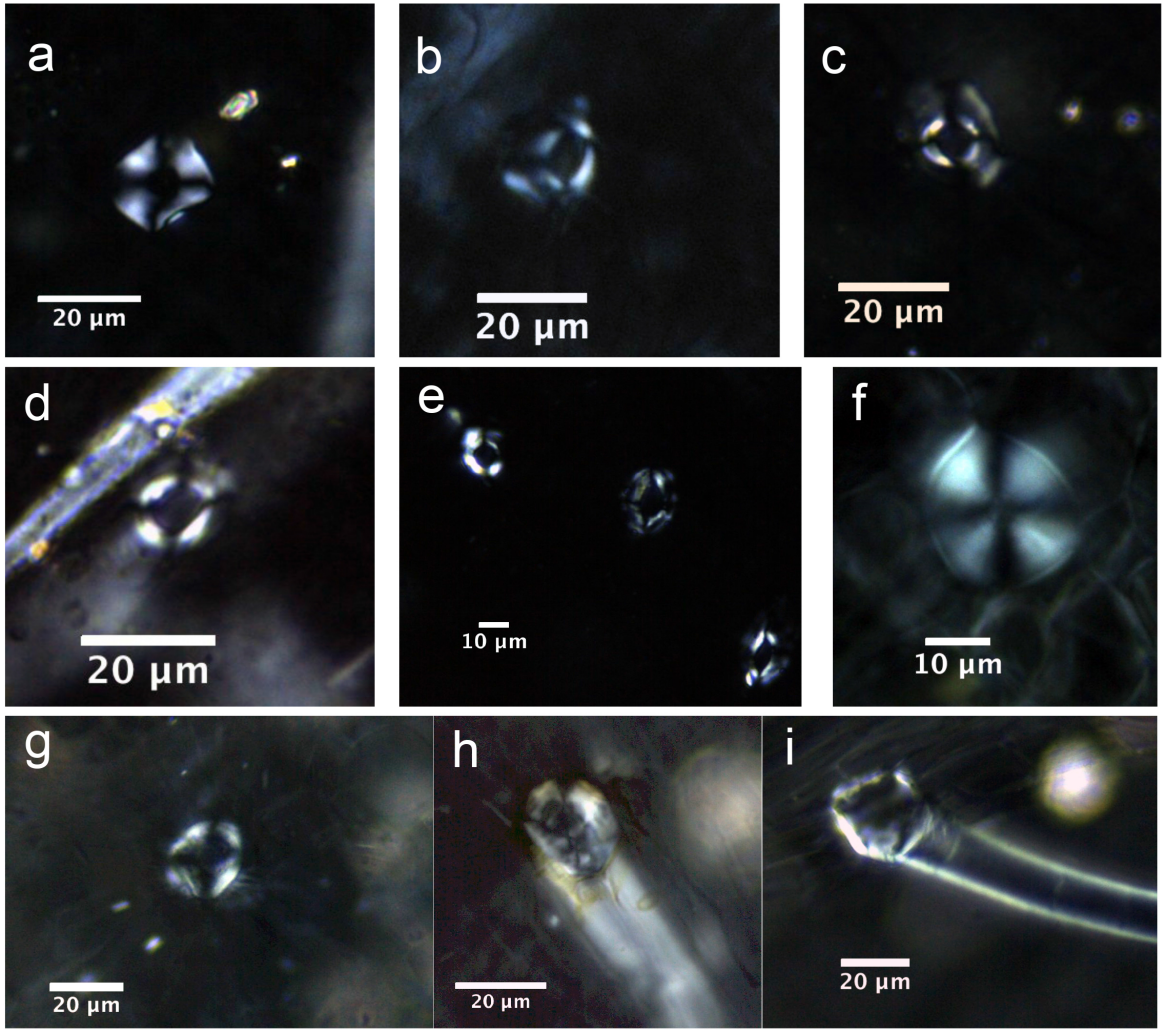
Morphological comparison of calcified trichome bases in *Camellia sinensis* L., Theaceae plants, and non-Theaceae plants. a) *Camellia sinensis* L. An'huihoukui; b) *Camellia sinensis* L. Liu'anguapian; c) *Camellia sinensis* L. Sichuanbaihao; d) *Camellia sinensis* L. Mengdingcha; e); *Camellia sinensis* L. Yeshengdayecha; f) *Camellia synaptica* S*.*; g) *Morus australis*; h) *Betula delavayi*; i) *Prunus mahaleb* var. *cupanian.*

Almost all of the leaves of the investigated plants contained six types of crystal (prisms, styloids, raphides, druses, crystal sands) in addition to calcified trichome bases, secondary crystals and concretions/intermediate crystals in mesophyll and vascular bundles. The three most common crystal types found were druses, prisms and calcified trichome bases. Druses exhibit the greatest size range, from tiny to relatively large in dimension. None to prominent cores were seen in druses in 39 species, scattered mostly in mesophyll. All prisms are cuboidal, although they vary somewhat in size between species. A calcified trichome base was observed mainly in tea, although a few species of non-Theaceae plants also exhibited this type. These three dominant crystal types were always accompanied by a somewhat amorphous crystalline form in 24 species. These aberrant forms, described as “intermediate crystals” in previous research, may be incompletely formed druses or prisms^2^. They occurred almost exclusively in vascular bundles, and seldom occurred in mesophyll. Tiny secondary crystals were common in tea; sphaerites were the most common, but tiny acicular crystals and/or styloids were also seen in many species. Concretions are comprised of many compacted mini-calcium oxalate crystals^9^; they were only found in two species of *Lycium* sp. and *Chenopodium album* and were large in diameter. Figures 1,2,3,4,5,6,7,8,9,10,11 show examples of these crystal forms, while Table 1 presents the distribution of crystal types in vascular bundles and mesophylls, for the various genera of the three types of plants.

### *C. sinensis* L

The crystals in this species are druses, calcified trichome bases, concretions/intermediate crystals, and tiny secondary crystals. The most common type in all 14 species is druses (Fig. 1). Druses have the smallest size range compared with those from other plants surveyed, from tiny to relatively large, and do not possess a dark core at the center. Small druses always occur in vascular bundles (Fig. 1m), and the larger ones always occur in large numbers in mesophylls (Fig. 1). The druses can be classified into two categories in terms of outline. The first category is dominant, and usually has sharp edges and corners (Fig. 1a, b, c, d, e, l, m). The second category usually has rounder outlines with obtuse edges and corners (Fig. 1f, g, h, i, j, k, o). Both types can occur in a single species.

Tiny secondary crystals, which are mainly spherical but are also accompanied by tiny acicular crystals and/or styloids, are scattered randomly in ordinary mesophyll cells in all 14 species (Fig. 1n). Small numbers of intermediate crystals occur in a somewhat amorphous crystalline form in 11 samples of tea. They occur mainly in vascular bundles (Fig. 2), and seldom in mesophyll.

The calcified trichome base occurred in all 14 species of tea (Fig. 3a). A higher magnification overview (Fig. 3b) reveals a round-oval-shaped base with a black and relatively sharply outlined hole. Each base is separated into four parts by four straight and regular cracks, similar to a regular extinction cross. This form of crystal is quite common in each root of the trichome in the leaf.

### Theaceae plants

Five crystal types were found in Theaceae plants, while prisms, styloids and raphides were absent. The most common type is druses in mesophyll. Fig. 4 reveals that the druses in mesophyll cells comprise two subtypes, A and B, of different size. Careful scrutiny reveals that subtypes A and B both have a contour with relatively less projection than the ones from tea, and almost always have a dark core at the center. Subtype A (Fig. 4b–e) consists of naked druses with small cores, in 4 species (Table 1), whereas subtype B (Fig. 4f–j) is surrounded by crystal sand. The crystal sands, plus one embedded druse, are overwhelmingly dominant, occurring in 9 species (Table 1). Occasionally, however, dominant embedded druses occur in association with a few single druses in *Camellia japonica* L. ‘Sun Song', *Camellia reticulate* L. ‘Snow Elegans', and *Camellia yunnanensis* Cohen Stuart var. *trichodarpa* Ming (Fig. 4f). A calcified trichome base occurred solely in two species, *Camellia synaptica* S. and *C. costei* Cevl. This type consists of a regular and straight cross extinction with a tiny core in the center, and is different to the one in tea (Fig. 4a). Intermediate crystals and secondary crystals are rare, occurring only in 3 and 5 species, respectively (Table 1).

### Non-Theaceae plants

Among the 17 species of common Non-Theaceae plants, all crystal types occur in varying abundance (Table 1). Druses are overwhelmingly dominant, occurring in 12 species, and as the sole type in *Dianthus chinensis* L. and *Portulaca oleracea* L. (Fig. 5). Most of them are crystallized inside mesophyll cells and exhibit sharply pointed ends and small to large cores in the center (Fig. 5). Prisms were next in abundance, occurring in 8 species, although they were not predominant in most of them. These prisms always have quadrangular and prismatic bodies and occurred along vascular bundles (Fig. 6). Calcified trichome bases were found in 6 species, and consist of two subtypes. The first subtype is characterized by a round-oval-shaped outline associated with several irregular cracks, and occurred in *Prunus mahaleb* var. *cupaniana*, *Corylus heterophylla* and *Betula delavayi* (Fig. 7a–c). The second subtype exhibits a round-oval-shaped base with straight cross extinction and only occurred in *Morus australis* (Fig. 7d).

Concretions were found in 3 of the 17 surveyed species, mainly in *Chenopodium album* and *Lycium* sp. They were large in diameter and occurred in mesophyll cells (Fig. 8). Intermediate crystals are possibly incompletely-formed druses or polymerized prisms occurring in mesophyll cells and vascular bundles in *Chenopodium album*, *Prunus mahaleb* var. *cupaniana* (Fig. 2b) and *Lycium* sp., whereas in *Morus australis* (Fig. 2c) they almost exclusively occurred in vascular bundles. Species with concretions and intermediate crystals also contain other types of crystal, including druses (Fig. 8a), crystal sands or secondary crystals (Fig. 8b) (Table 1); therefore they were never the sole crystal form.

Crystal sand was found only in 3 of the species surveyed; it was irregularly shaped and occurred in vascular bundles and mesophyll cells (Fig. 9a, b). Tiny secondary crystal sphaerites and rudimentary star-like crystal aggregates were found in 5 species, scattered randomly in mesophyll cells (Fig. 9c). Raphides, with sharply pointed tips and associated with tiny secondary crystals, occurred in *Epilobium tibetanum* Hausskn. Each raphide presumably occurs in a mesophyll cell (Fig. 9d).

## Discussion

In terms of tea identification, and specifically in the genus of *C. sinensis* L., biomolecular methods such as High Performance Liquid Chromatography (HPLC) are increasingly used because of their ability to detect unique biomolecular components, e.g. theanine, catechins, caffeine and gallic acid. However, it is difficult to extract them from tea residuals and therefore this approach is rarely used in archaeological research^28^.

As micro-mineral bodies, calciphytoliths (calcium oxalate crystals) will be formed only as specific crystal types each with a subset of crystal morphologies characteristic of a particular species. This implies that the cells and the genetics of the particular species forming these crystals control the morphology^5^. This provides us with the possibility of separating individual species based on the presence of identifiable crystals in different plants^29^. To date, however, investigation of the morphology of calcium oxalate crystals in Theaceae plants has been very limited. The results of our survey constitute both the first investigation of the foliar macropatterns in *C. sinensis* L., and a comparison of several Theaceae and common Non-Theaceae plants. Druses, calcified trichome bases, secondary crystals and intermediate crystals are by far the most common types of crystal in the various species of *C. sinensis* L. Druses and calcified trichome bases are relatively distinctive compared with the other types.

Druses in *C. sinensis* L. mainly scattered in mesophyll cells and vascular bundles, and as single crystals without dark core at the center. The size variation of druses in *C. sinensis* L. is the smallest (11.65 ± 3.64 μm), whereas the sizes of druses in Theaceae (39.17 ± 12.96 μm) and Non-Theaceae plants (21.39 ± 14.03 μm) are much more variable (Fig. 10).

The results of the discriminant analysis as a percentage of correct classification are presented in Table 2. A total of 89.2% of *C. sinensis* L., 75.1% of Theaceae plants are correctly classified. These encouraging results indicate that the size of druses has a strong tendency to polarize between *C. sinensis* L. and other Theaceae plants. Although the percentage of correct classification for non-Theaceae plants is as low as 44.2%, however, compared with druses in *C. sinensis* L., most druses in this group grow a dark core at the center (Fig. 4). This obvious character can help to distinguish between *C. sinensis* L. and Non-Theaceae plants in some extent.

In *C. sinensis* L., the calcified trichome base is more distinctive in terms of its configuration. A central hole associated with straight, regular and cross extinction-like cracks occurs in each base. A morphological comparison reveals that the shapes of the bases in *C. sinensis* L. are very different to those in Theaceae and Non-Theaceae plants (Fig. 11). Bases in Theaceae are few in number and they lack holes at the center, while most bases in Non-Theaceae plants exhibit several irregular cracks, which can be easily distinguished. Therefore, based on the foregoing, we conclude that the calcified trichome base can be used as a distinguishing characteristic of tea. However, in order to distinguish *C. sinensis* L. species from the others requires the presence of identifiable druses and calcified trichome bases: 1) single small druses without cores, usually 11.65 ± 3.6 μm in length/diameter; 2) a calcified trichome base associated with a central hole together with straight, regular and cross extinction-like cracks. The co-occurrence of these two types of calciphytolith in archaeological deposits would enable the identification of tea with a significant degree of confidence.

In general, we provide the first tentative morphological criteria for distinguishing tea from other plants by calciphytoliths (calcium oxalate crystals) and a basis for the identification of tea in archaeological remains, especially where macrofossil preservation is poor. Our findings may contribute to understanding the history of tea and plant exploitation. Nevertheless, further work is needed to confirm our conclusions; for example, the morphology of calciphytoliths in a wide range of plants, particularly species of *C. sinensis* L., will need to be analyzed in detail.

## Methods

We examined modern leaves from14 types of tea, including Green tea, Black tea, Oolong tea and White tea collected from a specialized tea market in Beijing, China. In addition, 14 species of Theaceae plant were obtained from Kunming Botanical Garden, Kunming, Yunnan Province, China; and 17 species of common non-Theaceae herby and woody plants from the Institute of Geology and Geophysics, Chinese Academy of Sciences, Beijing, China (see Table 1).

Leaves of most species were too large to be processed whole, and in these cases six to eight leaf pieces of dimensions ~1 × 1 cm or smaller were removed from various parts of the leaf and were processed using a bleaching technique modified from that of Lersten and Horner^2^: 50% aqueous alcohol (no time limit), distilled water (15 min), full-strength household bleach (5% sodium hypochlorite) (1–3 h, depending on leaf toughness), distilled water (15 min), ethanol dehydration series [50, 70, 95, 100 (2×), 1:1/100%:xylol–about 10 min for each step]. After transfer to two final changes of pure xylol (no time limit) the unstained leaf samples were mounted in permount, a coverslip was added, and the slides were stored horizontally for several days at room temperature to allow hardening of the mounting medium. Finished preparations were viewed in cross-polarized light at ×400 magnification to enhance crystal visibility. After scanning the preparations and determining the crystal macropatterns, representative regions were chosen to provide photographic images.

## Author Contributions

J.Z. and H.L. designed the research; J.Z., H.L. and L.H. collected samples and performed the research; J.Z. and H.L. contributed new reagents/analytic tools; J.Z. analyzed the data and prepared all figures and tables; J.Z. and H.L. wrote the paper. All authors discussed the results and reviewed the manuscript.

## Figures and Tables

**Table 1:** Crystal components within 45 samples of investigated plant leaves

	Idioblast components						Other		Idioblast location		
Taxon	Prism	Styloid	Raphide	Crystal sand	Druse	Concretions/Intermediate	Calcified trichome base	Secondary crystals	Vascular bundles	Mesophyll	
***C. sinensis* L.**											
An'huiHoukui					++nc		++	+	+	++	
Baihaoyinzhen					++nc	(+i)	++	+	+	++	
Fujianbaicha					++nc	+i	+	+	+	++	
Fujianlvcha					++nc	(+i)	+	+	+	++	
Liu'anguapian					++nc	(+i)	++	+	+	++	
Xinyangmaojian					++nc	+i	++	+	+	++	
Jinjunmei					++nc		++	+	+	++	
Sichuanbaihao					++nc	+i	+	+	+	++	
Sichuanqueshe					++nc	(+i)	++	+	+	++	
Zhuyeqing					++nc	+i	++	++	+	++	
Mengdingcha					++nc	+i	++	+	+	++	
Yeshengdayecha					++nc	(+)	+	+	+	++	
Tieguanyin					++nc	(+i)	(+)	+	(+)	++	
Longjing					++nc		+	+	+	++	
**Theaceae plants**											
*C.* *impressinervis*					++co					++	
*Sladenia celastrifolia*					++co	(+i)			+	++	
*C. crassipes*					++co					++	
*C. reticulata* L.‘Baozhucha'				+	++e					++	
*C. rubituberculata* Chang					++c			+		++	
*C. sasanqua* T. ‘Autumn Peony'				+	++e	(+i)			+	++	
*C. yunnanensis* Cohen Stuart var. *trichodarpa* Ming				+	++e +co			(+i)		++	
*C. reticulate* L. ‘Snow Elegans'				+	++e +co			(+i)		++	
*C.* *sasanqua* T. ‘Read Jade'				+	++e					++	
*C.* *japonica* L. ‘Sun Song'				+	++e +co					++	
*C. synaptica* S.					+ co	+i	++		+	++	
*C. costei* Cevl				+	++e		+	+		++	
*C.* *japonica* L. ‘Benihakari'				+	++e					++	
*C. reticulate* L. ‘Chuxiongcha'				+	++e			(+i)		++	
**Non-Theaceae plants**											
*Philadelphus schrenkii* Rupr.				+	++co	(+i)		+	+	++	
*Epilobium tibetanum* Hausskn.			++					+		++	
*Lespedeza bicolor* Turcz.	++	(+i)							++		
*Prunus mahaleb* var. *cupaniana*	+				++co	+i	+		+	++	
*Lycium* sp.	+			++		++c; +i				++	
*Chenopodium album*					++co	++c		+		++	
*Cynanchum komarovii* Al. Iljinski				+		+i			+	++	
*Dianthus chinensis* L*.*					++co					++	
*Malva sinensis*					++co			+	+	++	
*Magnolia sieboldii* K. Koch								++			
*Portulaca oleracea* L.					++co					++	
*Morus australis*		+			++nc	+i	+		+	++	
*Tilia tuan* Szyszyl.	++								++	+	
*Corylus heterophylla*	(+i)				++co		+		+	++	
*Zelkova schneideeeriana* Hand.-Mazz.	++				++co	(+i)			+ +	+	
*Salix ernesti*	+				++nc						
*Betula delavayi*	(+i)				++nc	(+i)	+			++	

Notes: ++ = predominant; + = present; (+) = rare; nc = no core; co = core; e = druses embed into crystal sands; i = intermediate crystals; c = concretions.

**Table 2:** Classification results of discriminant analysis for druse size

		Predicted Groups			
	Groups	1	2	3	Total
Original count	1	1715	0	207	1922
	2	29	1061	323	1413
	3	521	161	497	1179
%	1	89.2	.0	10.8	100.0
	2	2.1	75.1	22.9	100.0
	3	44.2	13.7	42.2	100.0

1: *C. sinensis* L.; 2: Theaceae plants; 3: Non-Theaceae plants.
